# A New Duty

**Published:** 1876-06

**Authors:** 


### A NEW DUTY.

THE "*Printers' Ink Fountain*," of Philadelphia, states, that an editor, "in consequence of ill health and other multifarious duties," withdraws from his office. We had been under the impression that it was the duty of people to keep well. Our general reason for thinking so was, that a sick man was fit for nothing but to be physicked, and as taking physic was a bad practice, it was wrong, and more, a very silly thing, for any one to place himself in a condition which unfits him for doing anything useful, and fits him only to do what is bad.

